# Renal Papillary Hyperplasia as a Cause of Persistent Asymptomatic Microhematuria

**DOI:** 10.1089/cren.2018.0060

**Published:** 2018-09-20

**Authors:** Ortwin Heißler, Stephan Seklehner, Claus Riedl

**Affiliations:** ^1^ Department of Urology, Landesklinikum Baden-Mödling, Baden, Austria.; ^2^ Paracelsus Medical University, Salzburg, Austria.

**Keywords:** microhematuria, asymptomatic, renal papillary hyperplasia, flexible ureterorenoscopy

## Abstract

Asymptomatic microscopic hematuria (AMH) is incidentally found during routine health screenings. In the clinical evaluation of persistent AMH imaging modalities, CT urography, MR urography, and retrograde pyelography are of diagnostic importance. In case of pathologic findings (e.g., contrast-filling defects), endoscopic evaluation is mostly performed. To our knowledge, we report the first case of a patient with persistent AMH caused by biopsy-proven renal papillary hyperplasia.

## Introduction

Asymptomatic microscopic hematuria (AMH) is defined as the presence of three or more red blood cells per high-power field visible in a properly collected urine specimen without evidence of infection. Microscopic hematuria is a common incidental finding during routine health screenings by general practitioners, with a prevalence of about 2%–31%.^1^

The most common causes of microscopic hematuria are urinary tract infection, benign prostatic hyperplasia, and urinary calculi. However, up to 5% of patients with microscopic hematuria are found to have a urinary tract malignancy. The workflow of persistent AMH includes, among others, a physical examination, a complete medical history, laboratory evaluation (complete blood count and coagulation studies), urinalysis with culture, urine cytology, a variety of imaging modalities (renal and bladder ultrasonography, CT urogram or magnetic resonance urogram, and retrograde pyelogram), and urethrocystoscopy. If imaging studies reveal contrast-filling defects in the upper urinary tract, it is essential to differentiate malignant from benign causes by flexible ureterorenoscopy (fURS) with biopsies. Nonmalignant causes of contrast-filling defects are radiolucent stones, debris, endometriosis, nephrogenic adenoma, mycetomas, malakoplakia, inflammatory pseudotumors, blood clots, and renal papillary hyperplasia.

In this article, we report the case of a patient who presented at our department with AMH caused by renal papillary hyperplasia.

## Case Report

A 30-year-old female Caucasian was referred by her urologist because of persistent asymptomatic microhematuria and a suspicious finding on CT scan. The patient was naive to medication except oral contraception. Family history for malignancies in the upper and lower urinary tract, as well as for stone disease, was negative.

She was normotensive without pathologic findings in the physical examination. Laboratory findings were within normal range. Urinalysis demonstrated blood in the urine without proteinuria or signs of infection. A pregnancy test was negative.

Renal- and bladder ultrasonography as well as the cystoscopy were without pathological findings.

**FIG. 1.**
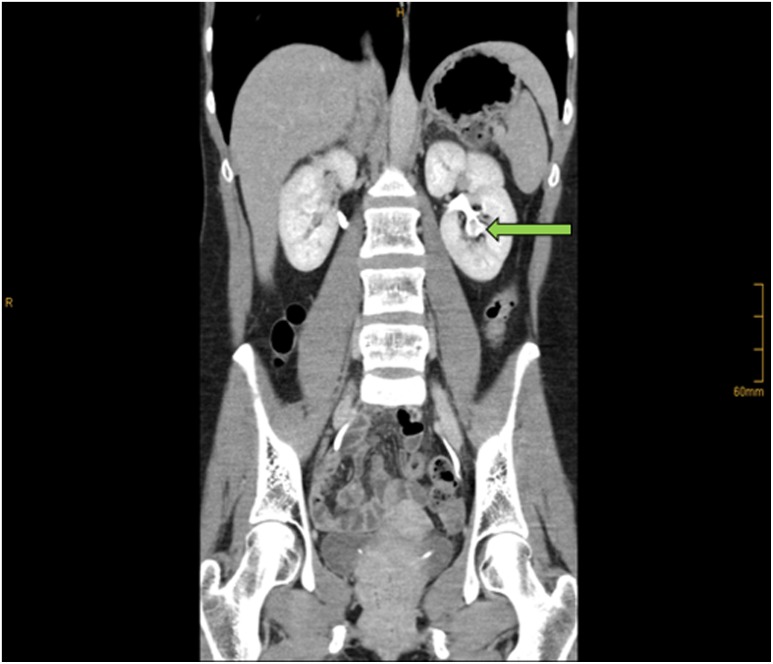
CT urogram showing filling defect in the left lower pole calix.

CT urography revealed a 5 mm filling defect in the left caudal calix (Fig. 1), so she was referred to our department to undergo retrograde pyelography and ureterorenoscopy to rule out upper urinary tract tumor.

**FIG. 2.**
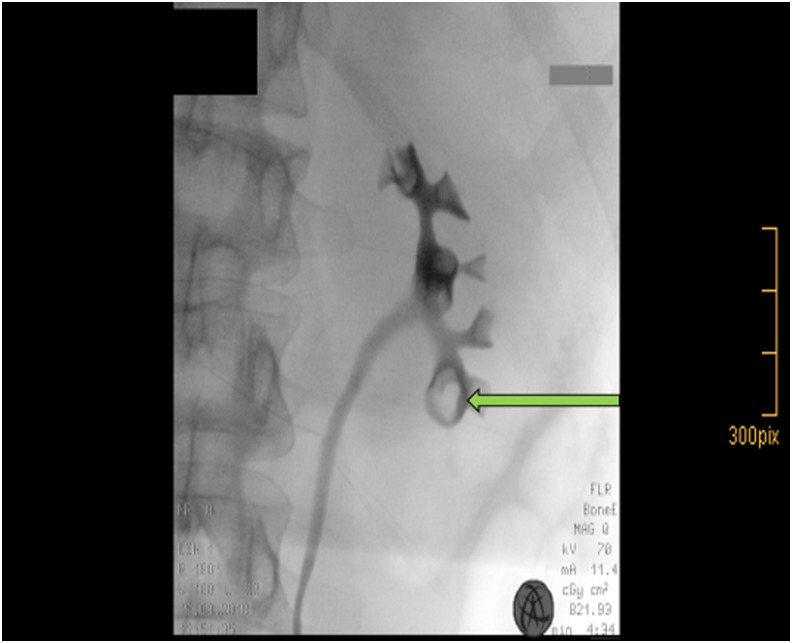
Filling defect in the left lower pole calix in retrograde pyelography.

**FIG. 3.**
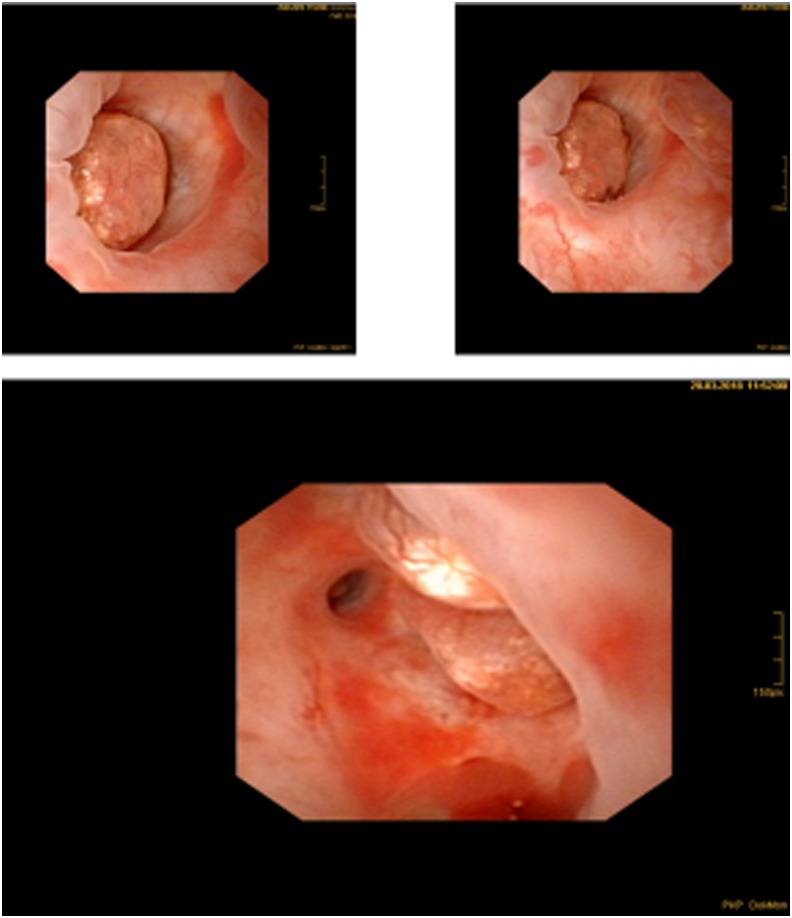
Hypertrophied papilla of the left lower pole calix.

The patient underwent cystoscopy with left-sided retrograde pyelography after sampling a urine cytology from the left renal pelvis. The urine cytology revealed papillary formations with nuclear atypia without malignancies (PAP III). The retrograde pyelogram showed the previously described contrast-filling defect in the caudal calix of the left kidney (Fig. 2). Because of a stenosis of the distal ureter, we placed a 7F Double-J stent and performed fURS 2 weeks later. The fURS was performed with a 9.9F video ureteroscope (Olympus, Tokio, Japan) and showed pathologic findings neither within the ureter nor within the renal pelvis. However, in two calices of lower pole, nodular calcified papillary structures were found (Fig. 3), which were both biopsied endoscopically and a 7F Double-J stent was placed again.

A second urine cytology from the left renal pelvis was taken during fURS, with similar results as obtained at the initial intervention. The histology analysis (including hematoxylin and eosin staining, Berliner Blau and CK20) of the biopsies showed a reactive urothelial hyperplasia without any malignancies.

After thorough communication with the patient about the benign cause of the AMH, we removed the Double-J stent without a subsequent surgery (e.g., ablation with a holmium-YAG laser). After 3 months, the patient was still asymptomatic and no changes were noted on CT scan with respect to contrast enhancement or the size of the lesion.

## Discussion

AMH is quite often found, but in people <35 years, no cause can be found in most cases. To our knowledge, our patient represents the first reported case of renal papillary hyperplasia as the cause of AMH.

The etiology of renal papillary hyperplasia is still unknown. Papillary hyperplasia and papillary adenoma, both considered benign, as well as papillary renal cell carcinoma have the same morphologic aspect on endoscopy. The only morphologic feature that may differentiate them is the size of the lesions: <15 mm for papillary adenoma and >15 mm for papillary renal cell carcinoma.^2^

Hyperplasia refers to an increase in the number of cell layers of the mucosal transitional cell epithelium. In the renal pelvis, the maximum number of cell layers is normally three to five, increasing to seven in the urinary bladder. The hyperplastic urothelium shows no cytologic atypia. Papillary hyperplasia has to be differentiated from low-grade papillary transitional cell carcinoma. Papillary hyperplasia lacks the characteristic fibrovascular core observed in papillary transitional cell carcinoma.^3^

Only few cases of renal papillary hyperplasia have been reported in the literature so far: available publications are from Lauret and colleagues^4^ in 1956, Moonen et al.^5^ in 1961 and Whitaker and colleagues^6^ in 1969. Their patients were all symptomatic (flank pain or macroscopic hematuria) and diagnosis of renal papillary hyperplasia was obtained after partial nephrectomy or radical nephrectomy, respectively.

Nowadays, the diagnosis and treatment can be achieved without such radical procedures. Türkvatan et al.^7^ reported on a 25-year-old woman with painless hematuria, in whom hypertrophic renal papillae were diagnosed by multidetector CT urography and static fluid MR urography without obtaining biopsies and lacking histologic verification.

Recently, Birk and coworkers^8^ published a case report with two female patients with persistent painless gross hematuria, who were both, as well as our patient, on hormonal contraception, and renal papillary hypertrophy was treated by holmium laser ablation, because in one patient the papilla was hypertrophic to the point of protrusion into the renal pelvis and in the other patient active oozing of blood was seen from the hypertrophic papilla.

We refrained to ablate the lesion as the patient was asymptomatic but chose a close follow-up regime with subsequent treatment if needed instead.

Owing to the lack of profound evidence, such as guidelines dealing with renal papillary hyperplasia, we suggest a close follow-up regime with regular visits every 4 months at the first year, including physical examination, ultrasonography, and urine cytology. CT or MRI studies may be performed twice a year. The latter may be prioritized because of the lack of radiation exposure.

Our follow-up strategy conforms to Türkvatan and Birk, who also suggested imaging studies twice a year.

In case of changes on imaging studies or if the patients becomes symptomatic (e.g., bleeding), laser ablation through retrograde intrarenal surgery will be the treatment of choice. At our department, Holmium:YAG and Thulium:YAG devices are prevalent, which have both been shown to be safe and effective.^9^ For both devices, 230 μm and 365 μm laser fibers can be used for ablation of tumors in the upper urinary tract with the corresponding laser settings. For instance, our Holmium:YAG laser (Auriga XL/4007, 120 W; StarMedTec GmbH, Starnberg, Germany) may be used with 1200 mJ and pulse of 18 Hz.

## Conclusion

Despite being a rare condition, renal papillary hyperplasia should be considered not only in symptomatic but also in asymptomatic patients presenting with microhematuria and a pelvicaliceal contrast-filling defect in imaging studies. As imaging modalities cannot safely distinguish nonmalignant from malignant conditions, further endourologic assessment including biopsy leads to definitive diagnosis and avoids unnecessary surgical treatment of this benign condition in asymptomatic patients.

## Abbreviations Used

AMH: asymptomatic microscopic hematuria
CT: computed tomography
fURS: flexible ureterorenoscopy
MRI: magnetic resonance imaging
